# Aggression and its association with childhood trauma in euthymic bipolar disorder patients

**DOI:** 10.1192/j.eurpsy.2023.1451

**Published:** 2023-07-19

**Authors:** A. Mellouli, R. Masmoudi, R. Ouali, F. Guermazi, I. Feki, R. Sellami, J. Masmoudi

**Affiliations:** Psychiatry A Department, Hedi Chaker University Hospital, Sfax, Tunisia

## Abstract

**Introduction:**

Aggression and negative behaviours are used to be present in individuals with bipolar disorder, who are sensitive to life events. Thus, many studies investigated the emergence of impulsivity and aggression in the developmental process and revealed its relationship with childhood adversities.

**Objectives:**

The aim of this study was to determine the relationship between childhood trauma and aggressive behaviour in euthymic patients with bipolar disorder.

**Methods:**

It was a cross-sectional descriptive and analytical study involving patients diagnosed with bipolar disorder and followed in the psychiatric department at the University Hospital ofSfax (Tunisia).

All subjects completed the Childhood trauma questionnaire (CTQ) and the Buss–Perry Aggression Scale (BPAS). Euthymiawas defined as a score on the Montgomery-Åsberg Depression Rating Scale (MADRS) not higher than 14 and by a score on theYoung Mania Rating Scale (YMRS)not higher than seven.

**Results:**

We included 35 patients. Their mean age was 46.69 ± 12.01 years with a sex ratio (M/F) =0.45. Most of them lived in urban areas (91.42%) and had a moderate socioeconomic level(88.57%).

The most frequent trauma type was physical neglect with 74.28%, followed by emotional abuse (42.85%), emotional neglect (42.85%), physical abuse (37.14%) and sexual abuse (31.42%).

The mean score of CTQ was 58.57 ±9.51. Theaverage total score of BPAS was 82.26 ±14.57.

The mean scores of subscales of BPAS were 25.49±4.59 for physical aggression, 13.74±3.51for verbal aggression, 19.14±6.22 for anger and 23.89±5.57 for hostility.

A statistically significant and positive correlation was determined between CTQ and BPAS (p=0.011). The score of BPAS was significantly correlated with physical abuse (p=0.003) and physical neglect (p=0.014).

**Conclusions:**

The relationship between CTQ and BGHA scores suggests the possibility that childhood trauma may be one determinant of aggression in patients with bipolar disorder. Considering the childhood trauma history in the evaluation of these patients may prevent their aggression and thus their psychosocial functioning.

**Disclosure of Interest:**

None Declared

